# Baseline total lesion glycolysis combined with interim positron emission tomography‐computed tomography is a robust predictor of outcome in patients with peripheral T‐cell lymphoma

**DOI:** 10.1002/cam4.3226

**Published:** 2020-06-18

**Authors:** Akihiro Kitadate, Kentaro Narita, Kouta Fukumoto, Toshiki Terao, Takafumi Tsushima, Hiroki Kobayashi, Yoshiaki Abe, Daisuke Miura, Masami Takeuchi, Youichi Machida, Kosei Matsue

**Affiliations:** ^1^ Division of Hematology/Oncology Department of Internal Medicine Kameda Medical Center Kamogawa Japan; ^2^ Department of Hematology, Nephrology, and Rheumatology Akita University Graduate School of Medicine Akita Japan; ^3^ Department of Hematology Graduate School of Comprehensive Human Sciences University of Tsukuba Tsukuba Japan; ^4^ Department of Radiology Kameda Medical Center Kamogawa Japan

**Keywords:** interim PET, PETCT, PTCL, TLG, TMTV

## Abstract

**Background:**

Peripheral T‐cell lymphoma (PTCL) represents a heterogeneous and rare subgroup of aggressive lymphomas that generally demonstrate poor clinical outcomes with conventional treatment. Since the prognosis of PTCL is heterogeneous, more accurate risk assessment, and risk‐adapted treatment strategies are required. In this study, we examined whether interim positron emission tomography (iPET)‐computed tomography (PET/CT) results can be combined with baseline volume‐based metabolic assessments including total metabolic tumor volume (TMTV) and total lesion glycolysis (TLG) for risk stratification in PTCL.

**Methods:**

The data of 63 patients with nodal PTCL, who had analyzable baseline PET/CT and iPET, were retrospectively reviewed. We calculated the baseline TMTV and TLG values. All iPET responses were analyzed using the Deauville 5‐point scale.

**Results:**

On univariate analysis, a prognostic index for PTCL (PIT) higher than 2 (hazard ratio [HR], 2.03; *P *= .026), high TMTV (>389 cm^3^; HR, 2.24; *P *= .01), high TLG (>875; HR, 3.77; *P *= .0005), and positive iPET (HR, 2.18; *P *= .009) were significantly associated with poorer progression‐free survival (PFS). On multivariate analysis, only high TLG and positive iPET independently predicted both poorer overall survival (OS) and PFS. A model combining TLG and iPET showed that patients with low TLG and negative iPET had superior outcomes, with a 5‐year PFS and OS of 72% and 90%, respectively. Conversely, both 5‐year PFS and OS for those with high TLG and positive iPET were 0%.

**Conclusions:**

In summary, TLG combined with iPET predicted survival in PTCL more accurately. This information may help in the development of risk‐adapted treatment strategies for PTCL.

## Graphical abstract

Recently, volume‐based positron emission tomography‐computed tomography (PET/CT) assessments such as total metabolic tumor volume (TMTV) and tumor lesion glycolysis (TLG) have been reported to be useful prognostic indicators for various cancers. In this study, we found that baseline TLG has stronger prognostic potential than baseline TMTV, and interim PET/CT response can be combined with TLG to improve risk stratification in peripheral T‐cell lymphoma.

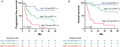

## 1 INTRODUCTION

Peripheral T‐cell lymphoma (PTCL) represents a heterogeneous subgroup of aggressive lymphomas with generally poor clinical outcomes on standard treatment.^1^ According to the WHO classification, the most common entities associated with PTCL are PTCL not otherwise specified (PTCL‐NOS), followed by angioimmunoblastic T‐cell lymphoma (AITL), and anaplastic large cell lymphoma (ALCL).^2^

The combination of cyclophosphamide, doxorubicin, vincristine, and prednisolone (CHOP) is the most frequently used first‐line treatment for patients with PTCL‐NOS, AITL, and ALCL.^3^ Recently, brentuximab vedotin in combination with cyclophosphamide, doxorubicin, and prednisolone has emerged as a new frontline treatment option for patients with previously untreated ALCL or other CD30‐expressing PTCL.^4^ However, except in the case of anaplastic lymphoma kinase (ALK)‐positive ALCL, the efficacy of CHOP therapy is not satisfactory, and most patients show poor prognoses.^1^ Therefore, some physicians initially treat fit young patients with CHOP therapy, followed by consolidative autologous stem cell transplantation (ASCT) during the first remission. However, recently published data do not support this treatment strategy for all patients with PTCL.^5^ This may be due to the heterogeneity of PTCL; thus, further study is needed to clarify which types of patients may benefit from this intensive strategy. That is, there is an urgent need for more accurate risk assessment and risk‐adapted treatment strategies for PTCL. With progress in the molecular understanding of PTCL pathogenesis, novel findings of genetic alteration have helped refine further classification of PTCL and appear to be useful for risk stratification. For example, it has been shown that PTCL‐NOS cases with a strong GATA3 expression show poor survival.^6^ In addition, recently published data also show that gene expression profiling could define biological and prognostic subgroups within PTCL‐NOS.^7^ However, risk stratification based on clinical parameters has not been fully developed.

Positron emission tomography‐computed tomography (PET/CT) using ^18^F‐fluorodeoxyglucose (FDG) has become an important imaging modality. PET/CT is routinely used for the staging and evaluation of treatment response in patients with malignant lymphomas, including PTCL.^8^, ^9^ Importantly, PET/CT performed during therapy (interim PET, iPET) has been found to have prognostic impact in various lymphoma subtypes, reflecting early treatment response.^10^, ^11^ In particular, the Deauville 5‐point scale (5‐PS), which uses iPET has become a promising parameter for the risk stratification of Hodgkin lymphoma,^12^ and PET‐guided risk‐adapted strategies have been developed accordingly.^13^ The prognostic impact of iPET has also been reported in PTCL.^14^, ^15^, ^16^ However, iPET is not commonly used as a treatment guide in clinical practice.

In addition to early response to treatment, baseline characteristics such as tumor burden and metabolic activity also significantly impact the outcomes. The baseline maximum standard uptake value (SUV_max_) is commonly used as a semiquantitative measurement. However, the prognostic value of SUV_max_ alone is limited,^17^, ^18^ as it represents a considerably small portion of a lesion, and lacks information on tumor burden, another factor important for prognosis. Therefore, volume‐based metabolic assessments including those of total metabolic tumor volume (TMTV) and total lesion glycolysis (TLG) have emerged as parameters with greater quantitative power. The TMTV is an estimate of the total tumor burden, and several studies have shown that it is predictive of clinical outcomes in various malignancies including malignant lymphoma.^19^, ^20^ TLG is calculated by multiplying the metabolic tumor volume (MTV) by mean SUV; thus, it is reflective of both the metabolic activity and the tumor burden. Previous reports have shown that baseline TLG values have prognostic importance in several cancers.^19^, ^21^ Moreover, recent reports suggest that TLG is a stronger predictor than TMTV in soft‐tissue sarcoma and primary mediastinal large B‐cell lymphoma (PMBL).^18^, ^22^

Although the role of TMTV analysis has been elucidated in various lymphomas including PTCL, little is known about the predictive value of TLG in PTCL.^19^ In this study, we investigated the predictive value of baseline TLG in addition to TMTV, and confirmed whether iPET results could be combined with TLG for risk stratification in PTCL.

## 2 MATERIALS AND METHODS

### 2.1 Patients

The data of patients with confirmed PTCL, who were consecutively treated between April 2006 and December 2018 in our center, were retrospectively analyzed. Patients included in this retrospective study met the following criteria: (a) confirmed histological diagnosis of PTCL, (b) presence of pretreatment PET/CT and iPET evaluation, and (c) receipt of anthracycline‐based chemotherapy as first‐line treatment. The diagnosis was confirmed in all cases by hematopathological review at our center. Clinical information obtained from all patients included those on age, sex, histopathological subtype, Eastern Cooperative Oncology Group Performance Status (ECOG PS), stage, bone marrow invasion, sites of extranodal infiltration, level of lactate dehydrogenase (LDH), Prognostic Index for PTCL (PIT),^23^ death, and relapse. This study protocol was approved by the Institutional Review Board before commencing this study. This study was carried out in accordance with the Declaration of Helsinki.

### 2.2 PET/CT parameters

FDG PET/CT scan was performed using dedicated PET/CT scanners (Discovery ST Elite Performance; GE Healthcare). The SUV was normalized to body weight and injected dose. The baseline SUV_max_ was measured in all detected lesion, and the highest FDG uptake was considered as the SUV_max_ of the patient. The TMTV was defined as the sum of the volumes of all lymphoma‐associated voxels with SUV of ≥2.5, as previously described.^24^ The TLG was calculated from the MTV and the mean SUV of all lesions. A semiquantitative analysis of the PET/CT scans for TMTV and TLG was performed using an open‐source software application Metavol (Hokkaido University).^25^ Bone marrow uptake was calculated only if there was focal uptake. iPET was defined as PET/CT which was performed after two to four cycles of induction chemotherapy. Deauville 5‐PS was used for assessment of iPET, with a score of 4‐5 reflecting positivity.^26^ We also analyzed quantitative SUV_max_ reduction between the baseline PET/CT and iPET by calculating the SUV_max_ decrease proportion (ΔSUV_max_). All quantitative and volumetric parameters were retrospectively analyzed in a blinded fashion by a nuclear physician.

### 2.3 Statistical analysis

Progression‐free survival (PFS) was defined as the duration from initial diagnosis till disease progression or death due to any cause. Overall survival (OS) was defined as the duration from diagnosis until death due to any cause. Survival fractions were calculated using the Kaplan‐Meier method and differences between groups were compared using the log‐rank test. Surviving patients were censored at the last follow‐up. The optimal cutoff values of the quantitative parameters (SUV_max_, ΔSUV_max_, TMTV, and TLG) were calculated by receiver operating characteristic (ROC) analysis. Cox proportional hazards regression models were used for multivariate analysis. *P* < .05 was considered statistically significant. Owing to the presence of strong correlations, the TMTV and TLG scores were considered in separate analyses. All statistical analysis was performed by GraphPad Prism 8 (GraphPad Software Incorporation) and R software v3.2.3.

## 3 RESULTS

### 3.1 Patient characteristics

**Figure 1:**
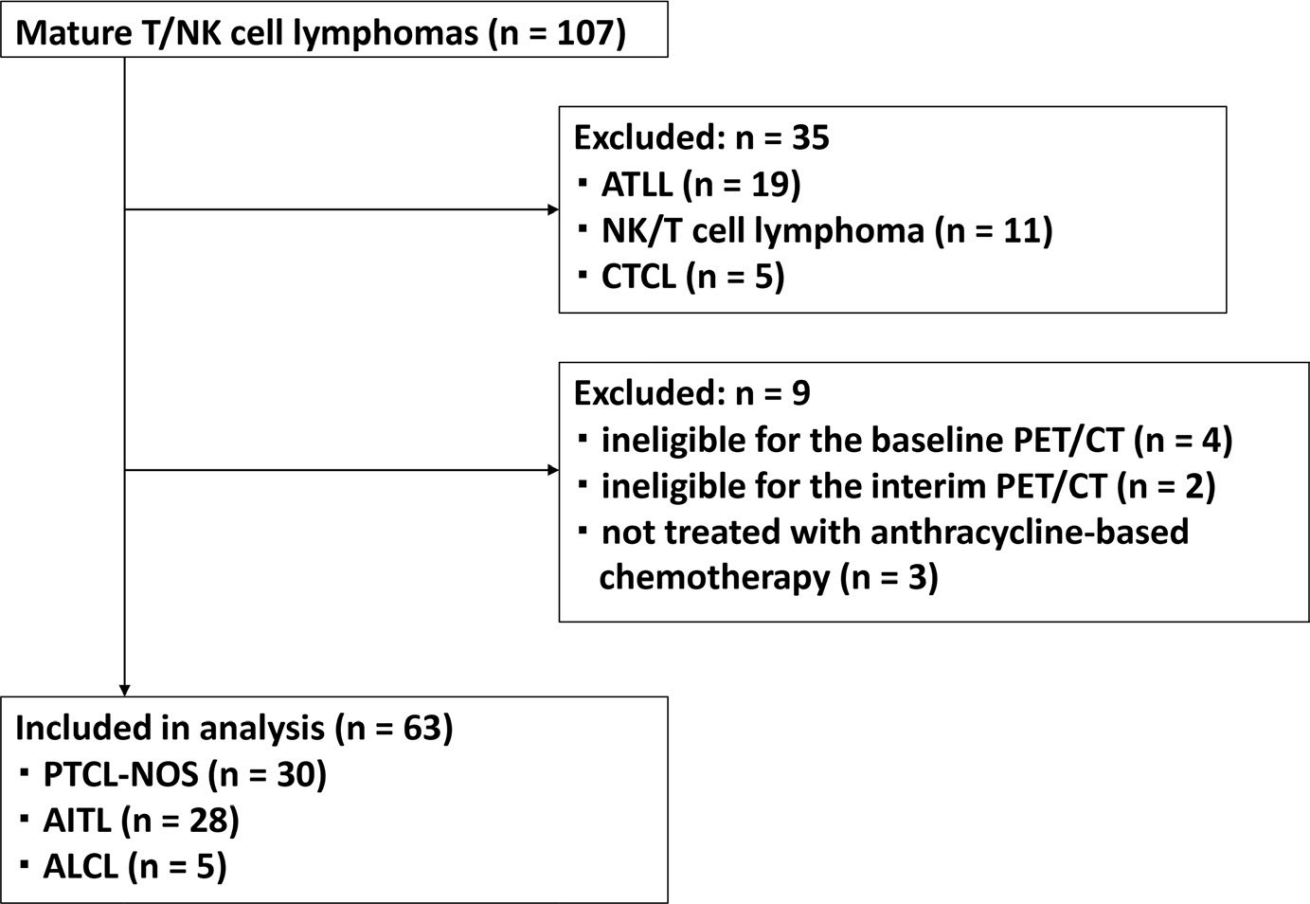
Flow diagram of patient selection. AITL, angioimmunoblastic T‐cell lymphoma; ALCL, anaplastic large cell lymphoma; ATLL, adult T‐cell leukemia/lymphoma; CTCL, cutaneous T‐cell lymphoma; PTCL‐NOS, peripheral T‐cell lymphoma not otherwise specified

**Figure 2:**
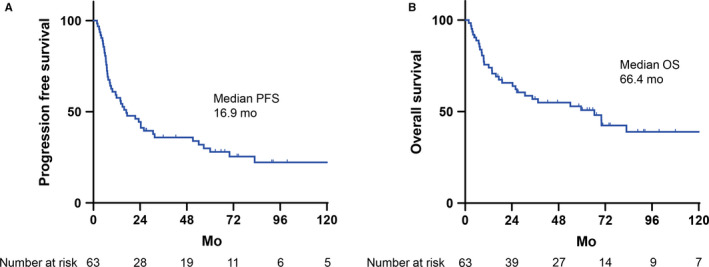
Kaplan‐Meier estimates of progression‐free survival and overall survival for the cohort. PFS (A) and OS (B) curves in the entire cohort. OS, overall survival; PFS, progression‐free survival

Among 107 patients with PTCL in our cohort, we excluded those with adult T‐cell leukemia/lymphoma (ATLL; n = 19), cutaneous T‐cell lymphoma (n = 5), and extranodal NK/T‐cell lymphoma (n = 11), owing to the associated different treatment strategies. In addition, we excluded three patients who did not receive anthracycline‐based chemotherapy as first‐line treatment, four who did not have analyzable baseline PET/CT results, and two who did not have analyzable iPET data (Figure 1). None of the patients were unable to undergo iPET due to disease progression. Finally, 63 patients, including those with PTCL‐NOS (n = 30), AITL (n = 28), ALK‐negative ALCL (n = 4), and ALK‐positive ALCL (n = 1), participated in this study (Table 1). The median age of these patients was 73 (range: 46‐88) years. CHOP or CHOP‐like chemotherapy was used for the majority of patients. Almost all patients underwent iPET after three cycles of chemotherapy. Consolidative stem cell transplantation, either autologous (n = 6) or allogeneic (n = 1), was performed in only seven (11%) patients, as the age of this cohort was relatively higher and only a minority were eligible for ASCT. After a median follow‐up of 35 months, the 5‐year PFS and OS for all patients were 30% and 51%, respectively (Figure 2A,B). The 5‐year PFS and OS were 29% and 31% for PTCL‐NOS, 26% and 65.4% for AITL, and 60% and 80% for ALCL, respectively. The other clinical parameters are described in Table 1. We then examined the prognostic impact of the baseline values of the clinical and biological parameters. On univariate analysis, sex, age, ECOG PS, LDH level, bone marrow invasion, and disease stage were not associated with poorer PFS or OS (Table 2). A PIT higher than two was predictive of poorer PFS (*P* = .026; hazard ratio [HR], 2.03; 95% confidence interval [CI], 1.08‐3.83) and OS (*P* = .03; HR, 2.21; 95% CI, 1.06‐4.60).

**Table 1:** Patient characteristics

Characteristics	Number of patients (%)
Age, y	
≤60	10 (16)
>60	53 (84)
Sex	
Male	34 (54)
Female	29 (46)
Diagnosis	
PTCL‐NOS	30 (48)
AITL	28 (44)
ALCL, ALK−	4 (6)
ALCL, ALK+	1 (2)
Ann Arbor stage	
Stage I‐II	9 (14)
Stage III‐IV	54 (86)
ECOG PS ≥2	13 (21)
Elevated LDH level	49 (78)
Bone marrow involvement	13 (21)
PIT	
0‐2	47 (75)
3‐4	16 (25)
First‐line chemotherapy	
CHOP/CHOP‐like	59 (94)
Others	4 (6)
Consolidative transplantation	
Autologous	6 (10)
Allogenic	1 (2)

Abbreviations: AITL, angioimmunoblastic T‐cell lymphoma; ALCL, anaplastic large cell lymphoma; ALK, anaplastic lymphoma kinase; CHOP, cyclophosphamide, doxorubicin, vincristine, and prednisone; ECOG, Eastern Cooperative Oncology Group; LDH, lactate dehydrogenase; PIT, Prognostic Index for Peripheral T‐cell lymphoma; PS, performance status; PTCL‐NOS, peripheral T‐cell lymphomas not otherwise specified.

**Table 2:** Univariate analysis of the factors predictive of survival

Parameter	N (%)	5‐y PFS (95% CI)	*P*	5‐y OS (95% CI)	*P*
SUV_max_			.768		.141
Low	28 (44%)	27% (12%‐46%)		61% (39%‐77%)	
High	35 (56%)	32% (17%‐48%)		44% (27%‐60%)	
TMTV			.01		.002
Low	27 (43%)	52% (31%‐69%)		75% (53%‐88%)	
High	36 (57%)	14% (5%‐28%)		33% (18%‐49%)	
TLG			.0005		<.0001
Low	21 (33%)	67% (40%‐83%)		80% (59%‐91%)	
High	42 (67%)	14% (6%‐27%)		29% (14%‐45%)	
Interim PET			.009		<.0001
Negative	38 (60%)	40% (24%‐56%)		74% (55%‐85%)	
Positive	25 (40%)	16% (5%‐33%)		17% (5%‐35%)	
ΔSUV_max_			.033		.006
>84%	34 (54%)	42% (24%‐59%)		70% (50%‐83%)	
≤84%	29 (46%)	17% (6%‐33%)		29% (14%‐47%)	
Age, y			.109		.053
≤60	10 (16%)	44% (14%‐72%)		89% (43%‐98%)	
>60	53 (84%)	27% (16%‐40%)		44% (29%‐57%)	
LDH			.597		.838
Normal	14 (22%)	42% (15%‐66%)		50% (21%‐74%)	
Increased	49 (78%)	27% (15%‐40%)		51% (36%‐64%)	
PS			.209		.108
0‐2	47 (75%)	31% (18%‐44%)		53% (38%‐66%)	
3‐4	16 (25%)	31% (10%‐55%)		46% (19%‐70%)	
BMI			.084		.631
Negative	50 (79%)	34% (21%‐48%)		53% (38%‐66%)	
Positive	13 (21%)	15% (3%‐39%)		43% (16%‐68%)	
PIT			.026		.030
0‐2	47 (75%)	35% (21%‐49%)		57% (41%‐70%)	
3‐4	16 (25%)	17% (3%‐39%)		33% (10%‐58%)	

Note

*P*‐values showing the level of significance in the univariate analysis (log‐rank test). SUV_max_, TMTV, and TLG were dichotomized using an optimized cutoff value. The optimal cutoff value determined using ROC curve analysis was 12.0 for SUV_max_, 389 cm^3^ for TMTV, and 875 for TLG.

Abbreviations: BMI, bone marrow invasion; CI, confidence interval; LDH, lactate dehydrogenase; OS, overall survival; PFS, progression‐free survival; PIT, Prognostic Index for Peripheral T‐cell lymphoma; PS, performance status; SUV_max_, maximum standard uptake value; TLG, total lesion glycolysis; TMTV, total metabolic tumor volume.

### 3.2 Baseline quantitative PET/CT parameters

**Figure 3:**
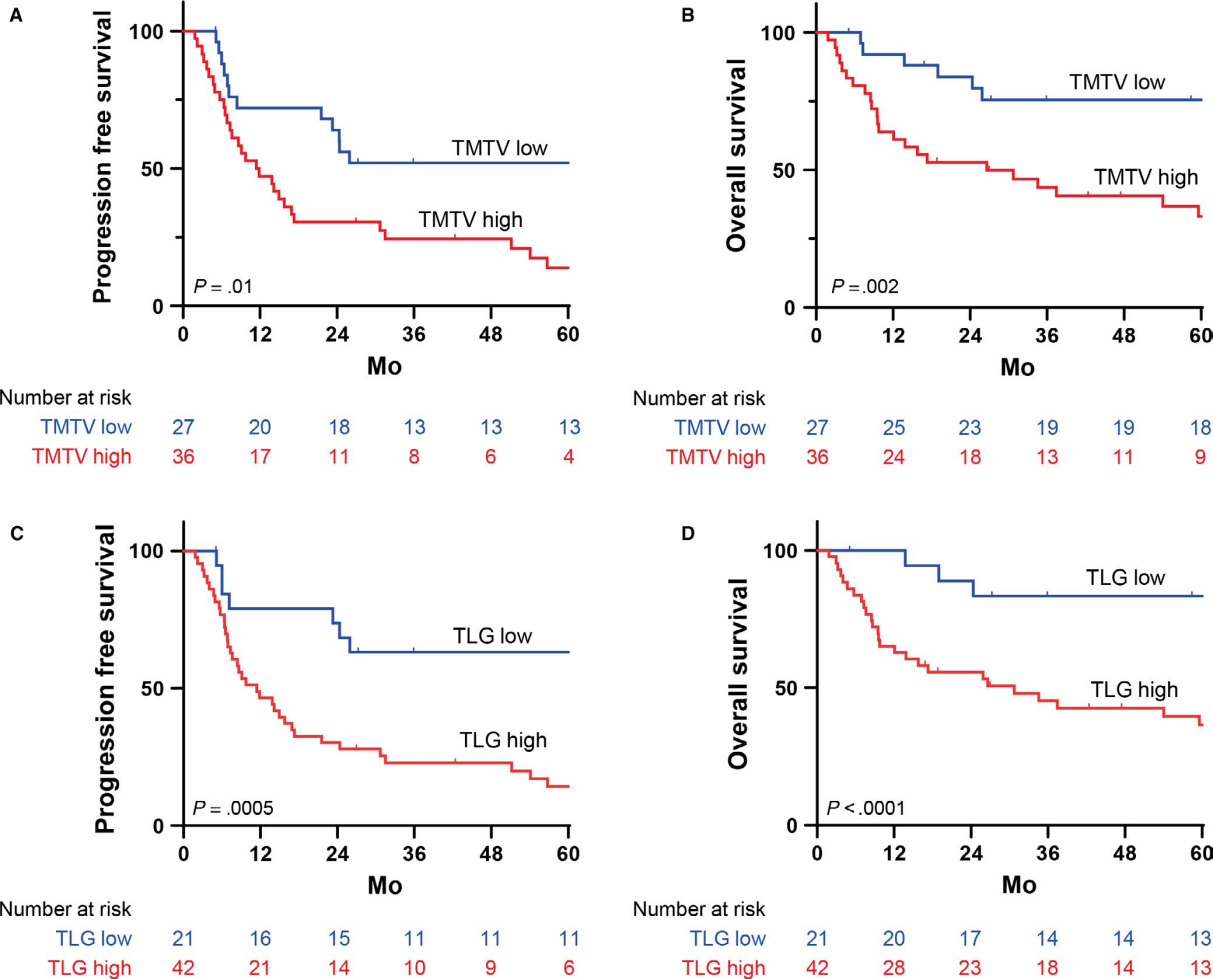
Comparisons of survival according to the cutoff value of TMTV and TLG. The baseline TMTV and TLG results were associated with both PFS (A,C) and OS (B,D), as determined by the log‐rank test. TMTV and TLG were dichotomized using an optimized cutoff value. The optimal cutoff value determined using receiver operating characteristic curve analysis was 12.0 for SUV_max_, 389 cm^3^ for TMTV, and 875 for TLG. OS, overall survival; PFS, progression‐free survival; SUV_max_, maximum standard uptake value; TLG, total lesion glycolysis; TMTV, total metabolic tumor volume

First, we examined the prognostic value of the baseline quantitative PET/CT parameters. The baseline PET/CT results were positive in all patients, and the median SUV_max_ was 13.1 (range, 2.6‐35.4). The baseline TMTV and TLG values were calculated for all patients; the median TMTV and TLG values were 423 cm^3^ (range, 21‐3012 cm^3^) and 1980 (range, 56‐21 400), respectively. The cutoff values with the highest sensitivities were 12.0 for SUV_max_, 389 cm^3^ for TMTV, and 875 for TLG. The PFS and OS were not significantly different between the low and high SUV_max_ groups. A high baseline TMTV value was significantly associated with poorer PFS (HR, 2.244; *P* = .01) and OS (HR, 3.358; *P* = .002) (Figure 3A,B). Moreover, high TLG baseline values were highly predictive of poorer PFS (HR, 3.767; *P* = .0005) and OS (HR, 4.722; *P* < .0001) (Figure 3C,D). Notably, patients with a low TLG value showed superior outcomes, with a 5‐year PFS rate of 65% and 5‐year OS rate of 80%. In contrast, those with a high TLG value had significantly worse prognoses, with a 5‐year PFS rate of 16% and 5‐year OS rate of 29%. There was no statistically significant difference between the histological subgroups in terms of SUV_max_, TMTV, and TLG.

### 3.3 iPET analysis

**Figure 4:**
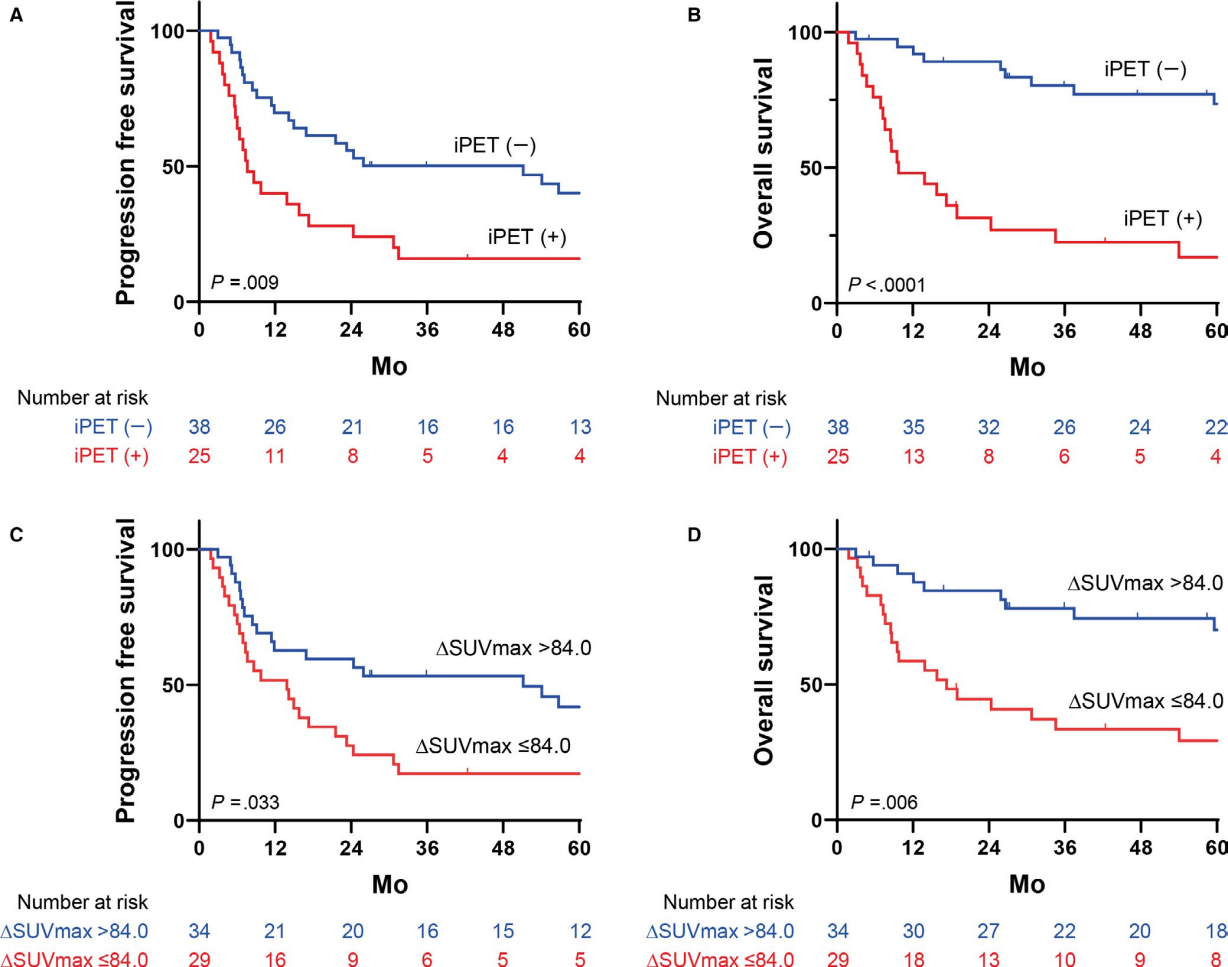
Kaplan‐Meier survival curves according to the iPET/CT results. iPET results were associated with both PFS (A) and OS (B), as determined by the log‐rank test. PET positivity was defined using a Deauville 5‐point scale, with a score of 4‐5 denoting positivity (^18^F‐FDG uptake higher than in the liver). ΔSUV_max_ can predict both PFS (C) and OS (D) in a subset of patients who had significant SUV_max_ reductions on iPET. The optimal cutoff value for ΔSUV_max_ determined using receiver operating characteristic curve analysis was 84% for both PFS and OS. ^18^F‐FDG, ^18^F‐fluorodeoxyglucose; CT, computed tomography; iPET, interim PET; OS, overall survival; PET, positron emission tomography; PFS, progression‐free survival; SUV_max_, maximum standard uptake value

Next, we confirmed the prognostic value of the iPET findings. The iPET results were negative in 38 of 63 (60%) cases. On univariate analysis, iPET positivity was predictive of poorer PFS (HR, 2.177; *P* = .009) and OS (HR, 4.931; *P* < .0001) (Table 2). Patients with negative iPET results showed good prognoses, with a 5‐year PFS rate of 40% and 5‐year OS rate of 74% (Figure 4A,B). In contrast, those with positive iPET results had poorer outcomes, with a 5‐year PFS rate of 16% and 5‐year OS rate of 17%. We then examined the prognostic value of ΔSUV_max_. The optimal cutoff value for ΔSUV_max_ was 84% for both PFS and OS. Patients with ΔSUV_max_ values higher than 84% showed significantly better PFS (HR, 1.885; *P* = .0033) and OS (HR, 2.566; *P* = .006) than those with ΔSUV_max_ values of 84% or lower (Figure 4C,D). We also examined the prognostic value of ΔMTV and ΔTLG; however, these had weaker predictive value than ΔSUV_max_. This may be due to the fact that the majority of participants showed a substantial reduction in the MTV after chemotherapy. In conjunction, these results indicate that early treatment response confirmed by iPET was also significantly associated with better prognoses.

### 3.4 Combining baseline TLG and iPET findings

**Figure 5:**
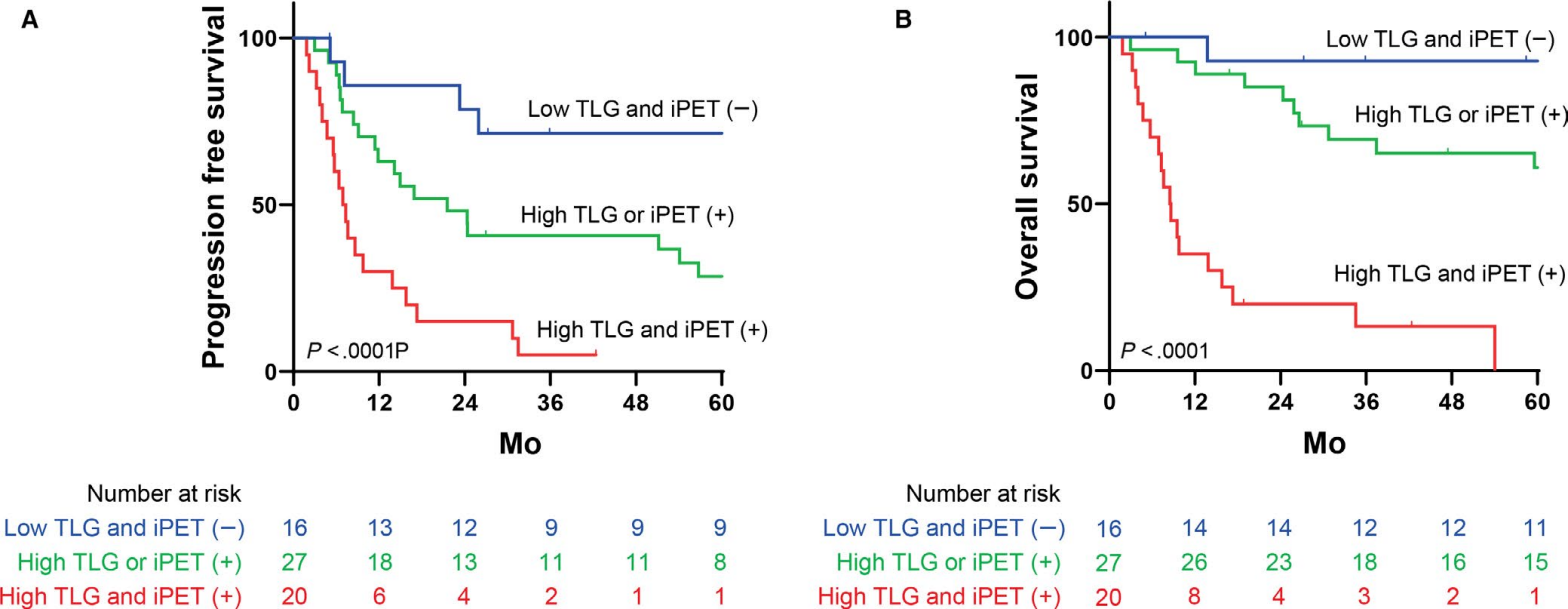
Combining baseline TLG with iPET. Kaplan‐Meier estimates of PFS (A) and OS (B) according to baseline TLG combined with interim PET. PET positivity was defined using a Deauville 5‐point scale, with a score of 4‐5 denoting positivity (^18^F‐FDG uptake higher than in the liver). The optimal cutoff value for baseline TLG determined using receiver operating characteristic curve analysis was 875. ^18^F‐FDG, ^18^F‐fluorodeoxyglucose; PET, positron emission tomography; PFS, progression‐free survival; OS, overall survival; TLG, total lesion glycolysis

On multivariate analysis testing TLG or TMTV with iPET results and PIT scores (Table 3), baseline TLG was a significant independent predictor for both PFS (HR, 3.158; 95% CI, 1.370‐7.278; *P* = .007) and OS (HR, 3.820; 95% CI, 1.543‐6.456; *P* = .004). The baseline TMTV showed a significantly unfavorable impact on PFS (HR, 2.048; 95% CI, 1.034‐4.055; *P* = .039), but not on OS (HR, 2.193; 95% CI, 0.927‐5.188; *P* = .074). These results suggest that TLG is a more useful predictor of both PFS and OS. As we hypothesized that baseline metabolic active tumor burden and poor response to initial treatment each contribute to poorer prognoses, we developed a prognostic model combining the baseline TLG and iPET results. As shown in Figure 5A,B, this model showed that patients with low baseline TLG values and negative iPET results had superior outcomes, with a 5‐year PFS rate of 72% and 5‐year OS rate of 90%. Notably, a majority of these patients with good prognoses (14/16, 87.5%) did not receive consolidative transplantation. Patients with high baseline TLG values and poor treatment response (iPET positive) had significantly worse prognoses, with a 5‐year PFS rate of 0% and 5‐year OS rate of 0%. Patients with high TLG values but good response (iPET negative) and low TLG values but poor response (iPET positive) showed intermediate prognoses, with a 5‐year PFS rate of 29% and 5‐year OS rate of 61%. There were direct correlations (*r* = .82; *P* = .001) between the groups stratified by ΔSUV_max_ and groups stratified by interim 5‐PS. The use of ΔSUV_max_ combined with baseline TLG was not superior to that of iPET combined with TLG.

**Table 3:** Multivariate analysis of the factors predictive of survival

	Parameter	Including TMTV		Including TLG	
		HR (95% CI)	*P*	HR (95% CI)	*P*
PFS	TMTV high	2.048 (1.034‐4.055)	.039		
	TLG high			3.158 (1.370‐7.278)	.007
	iPET positive	2.102 (1.137‐3.884)	.018	2.067 (1.123‐3.803)	.019
	PIT > 2	1.706 (0.864‐3.368)	.124	1.790 (0.933‐3.435)	.079
OS	TMTV high	2.193 (0.927‐5.188)	.074		
	TLG high			3.820 (1.543‐9.456)	.004
	iPET positive	4.614 (2.160‐9.857)	<.0001	4.914 (2.267‐10.65)	<.0001
	PIT > 2	1.994 (0.903‐4.403)	.087	1.631 (0.744‐3.574)	.222

Note

*P*‐values showing the level of significance in the multivariate Cox‐regression analysis. Owing to the presence of a strong correlation, TMTV and TLG scores were considered in separate analyses. TMTV and TLG were dichotomized using an optimized cutoff value. The optimal cutoff value determined using ROC curve analysis was 389 cm^3^ for TMTV and 875 for TLG.

Abbreviations: CI, confidence interval; HR, hazard ratio; iPET, interim positron emission tomography; OS, overall survival; PFS, progression‐free survival; PIT, Prognostic Index for Peripheral T‐cell lymphoma; TLG, total lesion glycolysis; TMTV, total metabolic tumor volume.

## 4 DISCUSSION

In this study, we found that baseline TLG is a reliable predictor of survival in PTCL patients. Notably, our data suggest that baseline TLG has stronger prognostic potential than baseline TMTV. Many previous studies that examined the quantitative parameters of PET/CT mainly focused on SUV_max_. As mentioned above, the prognostic value of SUV_max_ is limited as it indicates only the most active area of the tumor and may not reflect the overall metabolic tumor burden. Therefore, the evaluation of the overall tumor burden using TMTV was believed to overcome these limitations. However, the utility of TMTV is limited as it could not fully reflect the tumor metabolic activity. However, the TLG offers certain advantages in that it can reflect both the tumor metabolic activity and the entire tumor burden. As shown in this study, the SUV_max_ range in PTCL is considerably wide (2.6‐35.4); thus, TLG may be more useful in demonstrating metabolic active tumor volumes in such cases than in other lymphoma subtypes. Indeed, the multivariate analyses showed that TMTV was not an independent prognostic factor for OS, unlike TLG. These results indicate that TLG is a more useful predictor than TMTV: this finding was also reported in previous studies on sarcoma,^22^ lung cancer,^27^ and PMBL.^18^

Moreover, TLG in combination with iPET more accurately predicted survival in PTCL. Mehta‐Shah et al recently reported on the analysis of iPET and TMTV in PTCL,^28^ indicating that the use of TMTV allowed for the further classification of patients with favorable prognoses into subgroups of excellent and poor prognoses. Notably, favorable characteristics (low TMTV and negative iPET results) could be used to identify groups with a 5‐year event‐free survival rate exceeding 60%. Importantly, their cohort included patients who were treated with the intent to consolidate with ASCT. Indeed, a majority of patients (68%) underwent consolidation with stem cell transplantation. However, in our cohort, a majority of patients (89%) did not undergo consolidative transplantation. Nevertheless, in our study, favorable characteristics (low baseline TLG value and negative iPET results) showed excellent outcomes, with a 5‐year PFS rate of 72% and 5‐year OS rate of 90%. These results suggest that most patients with favorable values may not necessarily require ASCT for up‐front consolidation. Furthermore, patients with high TLG values and poor treatment response (iPET positive) showed extremely poor prognoses. As reported by Mehta‐Shah et al, patients with positive iPET results showed extremely poor prognoses. These results indicate that patients showing unfavorable characteristics (high TLG value and positive iPET) could not benefit from intensive chemotherapy such as ASCT. In such cases, allogenic transplantation should be considered in young and fit patients, as it has been demonstrated to be effective for relapsed/refractory PTCL.^29^ In elderly and unfit patients, alternative treatment strategies using novel agents such as monoclonal antibodies (eg, brentuximab vedotin) or histone deacetylase inhibitors (eg, romidepsin, and belinostat) may be considered.^30^

Our study has some limitations that must be acknowledged. First, it had a retrospective review design and a relatively small sample size. In addition, this study included different histological subtypes. Although this study, for the first time, showed that baseline TLG is a reliable predictor in PTCL, the aforementioned considerations also apply here. Therefore, further prospective multicenter studies are required to confirm these findings. Moreover, there is a discrepancy between the duration of PFS and that of OS in our study. Indeed, some of the relapsed patients were relatively young and underwent intensive chemotherapy and transplantation as salvage therapy (autologous, n = 3; allogenic, n = 2). Furthermore, patients in this study likely benefitted from improved salvage treatment and supportive care modalities, which contributed to longer survival. Importantly, patients with negative iPET results were often chemosensitive, even at the time of relapse, and these patients responded to salvage chemotherapy. Reflecting this, we also found a discrepancy between the duration of PFS and OS in iPET negative patients.

In summary, baseline TLG and iPET results are both independent prognostic factors in PTCL. Combining baseline TLG and iPET results can be used not only to identify groups of patients with favorable prognoses, but also extremely high‐risk patients that may benefit from more aggressive treatment or alternative treatment strategies earlier. This information could help in the development of risk‐adapted treatment approach for patients with PTCL showing variable prognoses.

## CONFLICT OF INTEREST

All authors have no conflict of interest to declare.

## AUTHOR CONTRIBUTIONS

AK designed the study, collected the data, performed the statistical analysis, and wrote the manuscript. KN and KF collected the data. TT, TT, HK, YA, DM, and MT provided patient care. YM interpreted the PET/CT images. KM supervised the study. All authors have reviewed and approved the manuscript.

## Data Availability

The data that support the findings of this study are available on request from the corresponding author. The data are not publicly available due to privacy or ethical restrictions.
